# A comparison of the validity and reliability between a digital radiographic imaging system and manual method in measuring the Cobb angle

**DOI:** 10.1186/1748-7161-8-S2-O20

**Published:** 2013-09-18

**Authors:** Min He Xiaohua, Jung KyeongAh, Ham HanSuk, Kim Ham JooHyun, Kim SungEun

**Affiliations:** 1 Palmer College of Chiropractic, Port Orange, FL, USA

## Background

The frontal plane Cobb angle measurement is an important clinical parameter in assessing scoliosis for therapeutic purposes. While manual measurement is still the predominantly used technique in diagnosing and treating scoliosis the use of digital X ray images in clinical practice is increasing[1].

## Purpose

The goal of this study was to conduct a clinical investigation quantifying scoliotic deformity with the Cobb angle to assess the intra- and inter-observer variability using manual and digital techniques.

## Methods

Twenty adolescents with scoliotic curvatures were chosen to participate in the study based on convenience, without predilection for gender, age, type or location. Images of the curvatures were examined by 15 trained observers to estimate the Cobb angle variability, as well as intra- and inter-observer variations. Each image was measured three times at a minimum interval of one week between measurements by each observer. For digital measurement, a special software x-maru viewer was developed to digitally reproduce the spine assessment; for manual measurement, traditional Cobb angle measurement was used. For comparisons between manual and digital measurements, Student’s t-test was used. To determine the inter-observer and intra-observer reliabilities, the intraclass correlation coefficients (ICC) were used. For the errors in measurement, 95% prediction limits were provided. For the manual measurement of Cobb angle, a mean ICC of 0.97 was determined for intra- and inter-observer reliability. For the digital measurements, a mean ICC value of 0.93 was determined for inter-observer reliability and a mean ICC value of 0.96 for intraobserver reliability.

## Results

Overall mean angle and standard deviation (95% confidence interval) were 16.97±0.94 for the manual method and 17.52±0.95 for the digital method (p>0.05). Inter-observer ICC was 0.926 for the manual method and 0.974 for the digital method. Digital measurement showed decreased intraobserver variability for many (11 of 15) of the radiographic parameters assessed. Likewise, digital measures indicated excellent correlation with the absolute values obtained with manual measurement for many (12 of 15) parameters.

## Conclusions and discussion

Although there was no statistical significance, the computer-assisted method is clinically advantageous and appropriate to assess the scoliotic curvature in the frontal plane using the Cobb method. Digital measurement showed improved measurement precision and good correlation with manual measurements for the majority of adolescent scoliosis parameters.
